# The Effect of Urban Shrinkage on Carbon Dioxide Emissions Efficiency in Northeast China

**DOI:** 10.3390/ijerph19095772

**Published:** 2022-05-09

**Authors:** Tianyi Zeng, Hong Jin, Zhifei Geng, Zihang Kang, Zichen Zhang

**Affiliations:** 1 Key Laboratory of Cold Region Urban and Rural Human Settlement Environment Science and Technology, Ministry of Industry and Information Technology, School of Architecture, Harbin Institute of Technology, Harbin 150006, China; jinhong@hit.edu.cn (H.J.); 20s034045@stu.hit.edu.cn (Z.K.); 20s034056@stu.hit.edu.cn (Z.Z.); 2 Business School, Ningbo University, Ningbo 315211, China; geng_zhifei@163.com

**Keywords:** CO_2_ emission efficiency, urban shrinkage, super-efficiency SBM model, mediating effect, northeast China

## Abstract

Climate change caused by CO_2_ emissions is a controversial topic in today’s society; improving CO_2_ emission efficiency (CEE) is an important way to reduce carbon emissions. While studies have often focused on areas with high carbon and large economies, the areas with persistent contraction have been neglected. These regions do not have high carbon emissions, but are facing a continuous decline in energy efficiency; therefore, it is of great relevance to explore the impact and mechanisms of CO_2_ emission efficiency in shrinking areas or shrinking cities. This paper uses a super-efficiency slacks-based measure (SBM) model to measure the CO_2_ emission efficiency and potential CO_2_ emission reduction (PCR) of 33 prefecture-level cities in northeast China from 2006 to 2019. For the first time, a Tobit model is used to analyze the factors influencing CEE, using the level of urban shrinkage as the core variable, with socio-economic indicators and urban construction indicators as control variables, while the mediating effect model is applied to identify the transmission mechanism of urban shrinkage. The results show that the CEE index of cities in northeast China is decreasing by 1.75% per annum. For every 1% increase in urban shrinkage, CEE decreased by approximately 2.1458%, with urban shrinkage, industrial structure, and expansion intensity index (EII) being the main factors influencing CEE. At the same time, urban shrinkage has a further dampening effect on CEE by reducing research and development expenditure (R&D) and urban compactness (COMP), with each 1% increase in urban shrinkage reducing R&D and COMP by approximately 0.534% and 1.233%, respectively. This can be improved by making full use of the available built-up space, increasing urban density, and promoting investment in research.

## 1. Introduction

As urban areas generally have high concentrations of population and industry, their carbon emissions constitute a major portion of global carbon emissions [1]. As the world’s largest emitter of CO_2_ [2], 70% of China’s CO_2_ emissions come from cities [3]. Therefore, whether the national policy on low carbon development and the carbon peak target can be achieved depends, to a large extent, on the CO_2_ emission efficiency (CEE) and potential CO_2_ emission reduction (PCR) of cities.

Many cities have developed climate action plans [4,5], primarily to meet long-term “low carbon” emission reduction targets set by national governments or interstate agreements [6]. However, most of these targets are only for cities that will continue to grow. As such, März et al. [7] raised the question of whether these mitigation strategies are applicable to cities under different development scenarios. The phenomenon of urban shrinkage is an inevitable dilemma faced by countries around the world at a certain stage of development [8]. China is experiencing rapid urbanization and facing huge inequality in regional development, with the large number of shrinking cities occurring as a result [9]. As the population is congregating more in the cities and mega-cities on the eastern coast, some small and medium-sized cities, as well as old industrial cities in transition, such as in the northeast, are experiencing population loss [10]. Cities in these regions are faced with persistently low energy efficiency or CO_2_ emission efficiency (CEE) [11,12], severely constraining the green and sustainable development of those cities. Northeast China was once a traditional heavy industrial base, but in recent years, it has become a growth depression area, contrary to China’s economic development, due to factors such as economic decline and population loss. This has led some scholars to refer to it as China’s “rust belt” region [13,14]. In addition, many Chinese cities are currently experiencing population contraction and spatial expansion, and urban shrinkage will further exacerbate urban vacancy and reduce urban compactness, thereby acting as a disincentive to promote energy efficiency [15].

CEE in today’s research mostly refers to linear planning models based on undesirable outputs to account for the efficiency of CO_2_ emissions at a national [16,17] or provincial level [18,19]. In the economic production process, labor, capital and energy inputs produce not only industrial products but also by-products such as CO_2_, i.e., undesirable outputs. A number of studies have used data envelopment analyses (DEA) for energy efficiency analyses [20,21]; however, the traditional DEA model focuses only on the desirable outputs of the economic activity process and ignores the undesirable outputs, which may lead to biased results [22,23]. Some scholars use improved models, such as the directed distance function model [24], slacks-based measure (SBM) model [25], super-efficient SBM model [26], etc. The results show that research and development expenditure, energy consumption structure, industrial structure, and economic activity level are the main factors affecting CEE in China [27,28]. In addition, market-oriented reforms [29], environmental regulations [30], and technology factors [18] have also been studied, while little attention has been paid to the effects of urbanization and urban shrinkage on CEE. Many studies have also failed to focus on the impact of mediating variables on energy efficiency.

The estimation of PCR is also key to the development of appropriate emission reduction policies and provides decision support to policymakers. CO_2_ emission reduction potential refers to the amount of CO_2_ emissions that can be avoided by implementing emission reduction technologies based on a combination of regional economic development and actual CO_2_ emissions [31]. Studies on CO_2_ emission potential estimation have used a variety of methods [32,33] among which the DEA efficiency variance estimation method has received the most attention. According to DEA theory, the efficiency frontier is composed of efficient decision-making units (DMUs), and the efficiency optimum is achieved by moving all DMUs to the efficiency frontier surface after resource reallocation, which is often regarded as PCR. DEA has been widely applied to estimate the CO_2_ reduction potential of different regions and sectors [34,35,36].

To compensate for the limitations of previous studies, this paper calculated the CEE and PCR of prefecture-level cities in northeast China for the 2006–2019 period based on the undesirable SBM model. Unlike previous studies, we used prefecture-level cities as the basic research unit; on this basis, the relationship between urban shrinkage and CEE was then analyzed. Finally, we built Tobit models and introduced mediating variables to analyze the factors affecting CEE. The results of this study will aid in the assessment of carbon reduction tasks in northeast China, guide policymakers in developing plans to improve CEE, and provide a scientific basis for a “smart contraction” with a view to carbon neutrality in Chinese cities.

## 2. The Calculation of CO_2_ Emission Efficiency

### 2.1. Study Area

**Figure 1:**
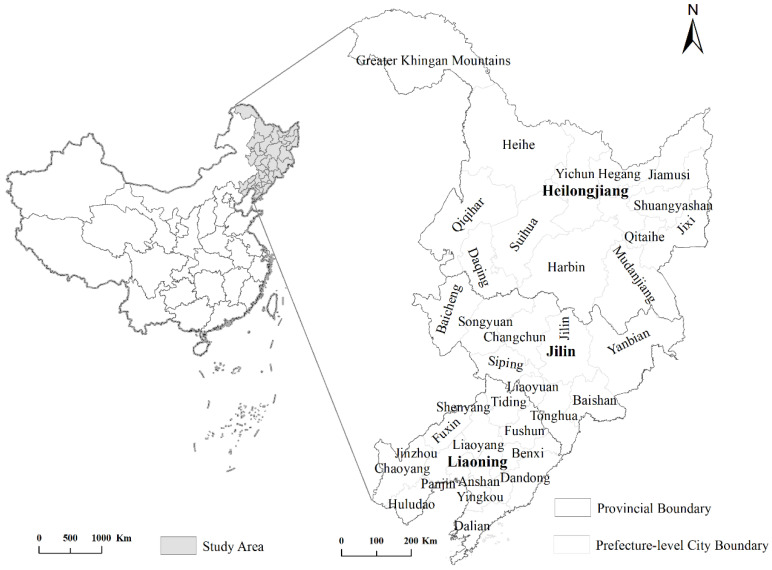
Study area in northeast China.

Northeast China is defined as the provinces of Liaoning, Jilin, and Heilongjiang. Liaoning province has 14 prefecture-level cities, Jilin province includes 8 prefecture-level cities and 1 autonomous prefecture, and Heilongjiang province manages 12 prefecture-level cities and 1 region (Figure 1). Due to the absence of statistics on the Daxinganling region, Suihua City, and the Yanbian Korean Autonomous Prefecture, a total of 33 prefecture-level cities are covered in this study. From 2006 to 2019, the population in the study area decreased from 97.19 million in 2006 to 95.77 million in 2019, a total decrease of 2.14 million and a population growth rate of −2.19%. Heilongjiang province contracted the most, with a population growth rate of −5.14%, while Liaoning and Jilin provinces experienced population growth rates of 0.05% and −2.20%, respectively. Yichun, Qitaihe, Jixi, Hegang, and Baishan experienced the most severe contraction, losing more than 10% of their populations. In research comparing the carbon emissions of cities in northeast China and the Yangtze River Delta, it was found that the rapidly shrinking group showed a continuous increase in CO_2_ emissions and a rising trend in their carbon intensity, and that the shrinking cities in northeast China have deviated from the development of a low carbon economy [10].

### 2.2. Data Source

For the calculation of CO_2_ emission efficiency, the capital stock, labor force, and total electricity consumption of each city in the northeast region were selected as the input factors, while the gross product and CO_2_ emissions of each city were used as the desired output and undesired output indicators. Table 1 presents the descriptive statistics of the input–output indicators:

(1) Capital stock (10^8^ dollars)—since the statistical yearbook does not explicitly provide data on the capital stock of each city, it is estimated by referring to the “perpetual inventory method”, which is commonly used in current studies [37].
(2) Labor input (10^4^ people)—the number of people employed in each city is taken from the provincial statistical yearbooks [25].
(3) Electricity consumption data (Total electricity consumption of primary, secondary and tertiary industries)—obtained from the statistical yearbooks of each province [38].
(4) Desirable output (10^8^ dollars)—the calendar year Goss Domestic Product (GDP) of each city is selected to express this.
(5) Undesirable output (million tons)—selected to be expressed in terms of carbon dioxide emissions for each city. Carbon dioxide data were collected from the Open-Data Inventory for Anthropogenic Carbon dioxide (ODIAC).

Most of the existing methods for calculating CO_2_ use “Method 1”, as described in the *2006* *IPCC* *(Intergovernmental Panel on Climate Change Guidelines for National Greenhouse Gas Inventories*, in which CO_2_ emissions are estimated based on the amount of fuel burned and default carbon emission factors [39]. However, precise and detailed energy consumption data for each region are only available at the inter-provincial level. Long time series energy consumption data specifically at the prefecture level are not fully available, so this paper uses the ODIAC open-source dataset of anthropogenic fossil fuel combustion CO_2_ produced by the Center for Global Environmental Research (CGER) [40].

ODIAC first introduced a combination of night-time lighting data and point source data from power plants to produce spatially gridded data on global fossil fuel CO_2_ emissions. To date, ODIAC has published several editions, such as ODIAC 2013a, ODIAC 2016, ODIAC 2018, and the latest, ODIAC 2020b. This paper uses the latest version ODIAC 2020b, which does not include international air and maritime carbon emissions and is monthly raster data with a spatial resolution of 1000 m [41]. The statistical results show that ODIAC 2020b can be used to effectively map the CO_2_ emissions of each prefecture-level city in the three northeastern provinces. Based on this dataset, we extracted spatially gridded data of carbon emissions in the northeast region from 2006 to 2019, synthesized them into annual data, and transformed them into the commonly used unit of CO_2_ measurement in China.

### 2.3. Super-Efficiency SBM Model Based on Undesired Output

DEA models are usually used to evaluate the technical efficiency scores between regions or between enterprises. With the concept of green development gaining increasing attention, scholars have gradually incorporated environmental constraints into the evaluation system. There are currently two DEA models for evaluating the efficiency of green outputs: the CCR (Charnes, Cooper, and Rhodes) or BCC (Banker, Charnes and Cooper) model with directional vectors, and the SBM model that considers undesirable outputs. The traditional CCR model is also known as the radial and angular model, where the “radial” requirement overestimates the efficiency of the unit being evaluated, while the “angular” assumption is not consistent with objective reality [42]. In order to overcome the radial and angular problems of traditional DEA models, Tone [43] constructed a new model based on a slack variable measure, i.e., the SBM model, which is a non-radial and non-angular DEA model. Tone [44] went further to include undesirable outputs in the evaluation model, and thus, constructed the undesired output SBM model. Compared to traditional data envelopment models (DEA), SBM models based on undesirable outputs can solve the problem of input–output slackness as well as the problem of efficiency analysis [45].

Assume that there are *n* decision units, each of which contains three elements: inputs, desirable outputs, and undesirable outputs, represented by the vector (*X*, *Y*, *B*), respectively, where the input, desired output, and undesired output vectors satisfy: *X* = (*x_ij_*) ∈ *R_m_*_×*n*_, *Y* = (*y_kj_*) ∈ *R_s_*_1×*n*_, and *B* = (*b_pj_*) ∈ *R_s_*_2×*n*_, so that if *X* > 0, *Y* > 0, and *B* > 0, m denotes the number of input variables, *s*_1_ denotes the number of desirable output, *s*_2_ denotes the number of undesirable outputs and *n* denotes the number of contemporaneous decision units (number of cities). The production possibility set is represented as:

$$P=\{\left(x,\;y\right)|x\geq \;X\Lambda ,\;y\leq \;Y\Lambda ,b\geq \;B\Lambda ;\;\Lambda \geq \;0\}$$

where $\Lambda =\left[\lambda_{1},\lambda_{2},\ldots ,\lambda_{n}\right]\in R^{n}$ denotes the vector of weight coefficients, and the three inequalities in the *P* function indicate that the actual input level is greater than the frontier level, the actual desired output is less than the frontier output level, and the actual undesired output is greater than the frontier level, respectively. The DMU (*x*_0_, *y*_0_, *b*_0_) is evaluated using the SBM model with undesirable outputs as shown in Equation (1):

(1) $$\rho =min\frac{1-\frac{1}{m}\sum_{i=1}^{m}\frac{s_{i}^{x}}{x_{i0}}}{1+\frac{1}{s_{1}+s_{2}}\left(\sum_{k=1}^{s_{1}}\frac{s_{k}^{y}}{y_{k0}}+\sum_{l=1}^{s_{2}}\frac{s_{l}^{b}}{b_{l0}}\right)}\;\;s.t.\;x_{i0}=\overset{n}{\underset{j=1}{\sum }}\lambda_{j}x_{j}+s_{i}^{x},\;\forall i;\;\;y_{k0}=\overset{n}{\underset{j=1}{\sum }}\lambda_{j}y_{j}-s_{k}^{y},\;\forall k;\;b_{l0}=\overset{n}{\underset{j=1}{\sum }}\lambda_{j}b_{j}+s_{l}^{b},\;\forall l;\;s_{i}^{x}\geq 0,\;s_{k}^{y}\geq 0,\;s_{l}^{b}\geq 0,\lambda_{j}\geq 0;\;\forall i,j,k,l;$$

where *s^x^* ∈ *R^m^* and *s^b^* ∈ *R^s^*^2^ represent the excess of inputs and undesirable outputs, respectively, while *s^y^* ∈ *R^s^*^1^ represents the deficiency of desirable outputs; ρ represents the CO_2_ emission efficiency of the decision unit; and *m*, *s*_1_, and *s*_2_ represent the number of variables for inputs, desirable outputs, and undesirable outputs, respectively. Equation (1) satisfies the assumption of constant returns to scale. Based on a comprehensive assessment of the level of economic development in northeast China in the observation period, it is believed that constant returns to scale are more in line with the actual situation in the region, so increasing or decreasing returns to scale will not be considered.

When ρ=1, which means *s^x^* = 0, *s^y^* = 0, and *s^b^* = 0, the DMU is efficient; and when ρ<1, which means there is non-zero relaxation $\left\{s\neq 0;\;\forall x,y,b\right\}$, the DMU is non-efficient and there is room for improvement. In line with the traditional DEA model, the efficiency value of the SBM model can only remain in the interval [0,1] and the efficient DMU takes the value of 1, while areas less than 1 are considered to be in an inefficient state. Therefore, we cannot compare efficient DMUs. In order to solve the problem of the incomparability of efficient regions, super-efficiency models are usually used. Compared with the traditional DEA super-efficiency models, the SBM super-efficiency model is slightly more complicated, as it does not simply add the restriction that *j* ≠ 0 [46]. Furthermore, the super-efficiency SBM model can only calculate efficient DMUs, but not inefficient ones. Therefore, in order to derive comparable values for all DMUs using the non-expectation SBM model, the efficient areas need to be calculated again using the super-efficiency SBM model with non-expectation outputs. Thus, the CO_2_ emission efficiency values in this paper are the combined results of the two models.

We recalculate the efficient DMU (*x*_0_, *y*_0_, *b*_0_) by referring to the undesirable output super-efficiency SBM model [47]. The formula is shown in Equation (2):

(2) $$\rho =min\frac{1+\frac{1}{m}\sum_{i=1}^{m}\frac{s_{i}^{x}}{x_{i0}}}{1-\frac{1}{s_{1}+s_{2}}\left(\sum_{k=1}^{s_{1}}\frac{s_{k}^{y}}{y_{k0}}+\sum_{l=1}^{s_{2}}\frac{s_{l}^{b}}{z_{l0}}\right)}\;s.t.\;x_{i0}\geq \;\overset{n}{\underset{j=1,\neq 0}{\sum }}\lambda_{j}x_{j}-s_{i}^{x},\;\forall i;\;\;y_{k0}\leq \;\overset{n}{\underset{j=1,\neq 0}{\sum }}\lambda_{j}y_{j}+s_{k}^{y},\;\forall k;\;b_{l0}\geq \;\overset{n}{\underset{j=1,\neq 0}{\sum }}\lambda_{j}b_{j}-s_{l}^{b},\;\forall l;\;1-\frac{1}{s_{1}+s_{2}}\left(\overset{s_{1}}{\underset{k=1}{\sum }}\frac{s_{k}^{y}}{y_{k0}}+\overset{s_{2}}{\underset{l=1}{\sum }}\frac{s_{l}^{b}}{b_{l0}}\right)>0;\;s_{i}^{x}\geq 0,\;s_{k}^{y}\geq 0,\;s_{l}^{b}\geq 0,\lambda_{j}\geq 0,\;\forall i,j,k,l;$$

It should also be noted that $s^{x},s^{y},s^{b}$ in Equation (2) is not a slack variable in the theoretical sense, and we still use the slack variable calculated in Equation (1) when calculating the excess and deficit of inputs, undesirable outputs, and desirable outputs.

## 3. Research Design

### 3.1. Modelling

Based on the previous analysis of the mechanism of urban contraction on CEE, we propose the following hypotheses:

**Hypothesis** **1.** *Urban contraction can have a direct impact on CEE.*

**Hypothesis** **2.** *Urban contraction suppresses CEE by reducing R&D.*

**Hypothesis** **3.** *Urban contraction suppresses CEE by reducing COMP.*

Since the CEE is non-negative, if least squares are used, the deviation of the parameters will be inconsistent. Tobin proposed the Tobit model [48], which is a regression analysis of the dependent variable, and when the dependent variable is finite, the use of the Tobit model is appropriate. In this study, the following econometric model was constructed to test the impact of urban shrinkage on CEE:

(3) $$CEE_{it}=\beta_{0}+\beta_{1}Shrink_{it}+\beta_{2}X_{it}+\varepsilon_{it}$$

where *i* and *t* denote city section units and corresponding years, respectively; Shrink represents the core explanatory variable urban shrinkage; *X* is a set of control variables; *β* denotes the parameter to be estimated; and *ε* is a random disturbance term.

The chosen mediating variables were R&D expenditure as a proportion of government expenditure (R&D) and urban population density (COMP), which capture the level of technological innovation and urban compactness of the city, respectively. In order to test Hypotheses 2 and 3, the following mediating effect model was constructed:

(4) $$CEE_{it}=\lambda_{0}+\lambda_{1}Shrink_{it}+\lambda_{2}M_{it}+\lambda_{3}X_{it}+\zeta_{it}$$

(5) $$M_{it}=\gamma_{0}+\gamma_{1}Shrink_{it}+\gamma_{2}X_{it}+\upsilon_{i}$$

where *M* denotes the mediating variable; *ζ* and *υ* both denote random error terms; and the other variables have the same sign as above.

This study used a stepwise test to verify whether urban shrinkage affects CEE through the mediating channel of technological innovation capacity and urban compactness. The significance of the coefficient *β*_1_ in Equation (3) was first tested, and if significant, the coefficient *γ*_1_ in Equation (5) and the coefficient *λ*_2_ in Equation (4) were tested for significance in turn, and if both were significant, this indicated the existence of a mediating effect.

### 3.2. Index Selection and Variable Description

We used urban shrinkage as the core explanatory variable, according to Murdoch al. [49]:

(6) $$Shrink_{it}=-\ln\left(\frac{pop_{it}}{pop_{it_{0}}}\right)$$

where pop denotes the number of people registered in the city at the end of the year and t_0_ is the initial year. The negative sign is introduced into the formula, which means that when the population moves out ($\frac{pop_{it}}{pop_{it_{0}}}\;$ < 1), the city experiences a contraction ($Shrink_{it}\;$ > 0), and the higher the number of people moving out, the larger the contraction indicator, meaning that the city is contracting more.

The expansion of built-up areas is a spatial manifestation of urbanization. This study also considers the impact of changes in urban built-up areas on carbon emissions [50]. We used the average annual rate of expansion (V) [51] and expansion intensity index (EII) [52] to measure the urban built-up area expansion:

(7) $$V_{it}=\left(\sqrt[{}]{\frac{S_{it}}{S_{it_{0}}}}-1\right)\times 100\%$$

(8) $$EII_{it}=\frac{S_{it}-S_{it_{0}}}{TLA}\times 100\%$$

where *S* is the built-up area of the city and *TLA* is the urban area.

In addition, we selected urban road area per capita (Road) and green space area per capita (Green) as the influence variables of built-up areas on CEE, since urban green space systems can reduce the carbon emission of traffic trips by guiding green trips (walking and cycling) [53,54], and road network density has a significant negative overall effect on the carbon emission of different types of trips [55]. The following socio-economic indicators were also selected as control variables: GDP per capita (GDPP) [56] and industrial structure (the ratio of secondary industry GDP to total GDP) (IS) [57]. Table 2 presents the descriptive statistics of the variables.

## 4. Calculation Results and Analysis of Carbon Emission Efficiency

The correlation between inputs and outputs was analyzed. The correlation coefficient showed a positive correlation at the 1% level of significance (Table 3), indicating that the more inputs there are, the more outputs there are. In particular, the relationship between GDP and CO_2_ showed a significant positive correlation, illustrating that the two sides influence each other.

**Figure 2:**
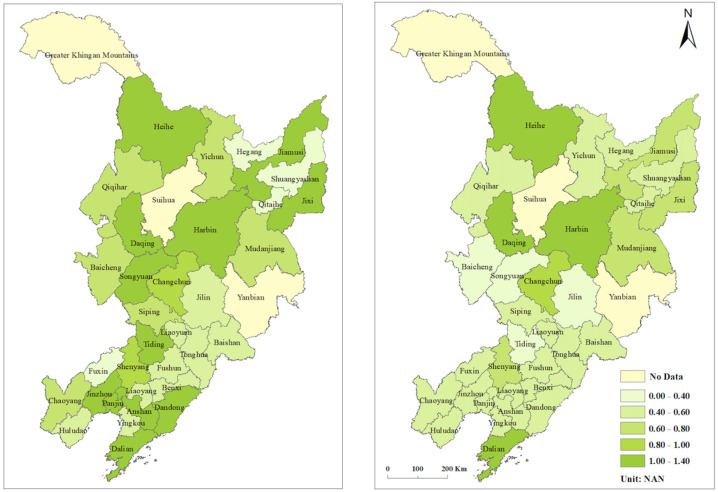
Carbon dioxide (CO_2_) emission efficiency of prefecture–level cities in northeast China.

**Figure 3:**
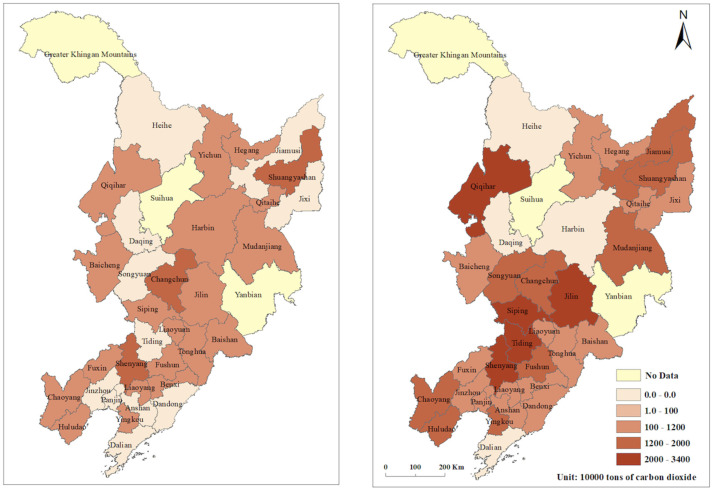
Potential carbon dioxide emission reduction (PCR) of prefecture–level cities in northeast China.

**Figure 4:**
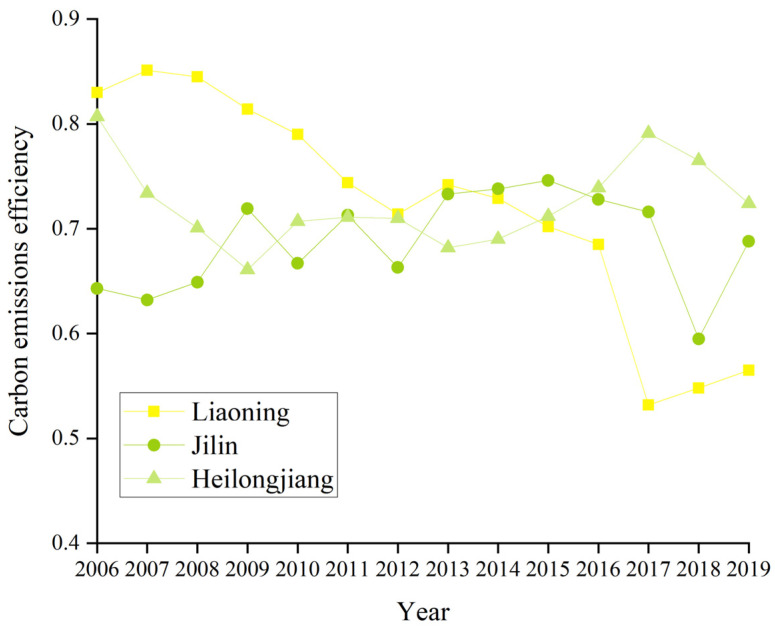
CO_2_ emission efficiency in northeast China from 2006 to 2019.

Based on the super-efficiency SBM model of undesired output, we calculated the CEE (Figure 2) and PCR (Figure 3); the results are shown in Table A1 and Table A2. In 2019, the average CEE of the cities in the northeast was 0.601, a 23% decrease compared with 0.777 in 2006, and the overall trend of CEE has continued to be low. Heilongjiang province had the highest CEE at 0.724, followed by Jilin province with a CEE of 0.688, while Liaoning province was at the bottom with the lowest CEE of 0.565. Overall, there was a downward trend in CEE from 2006 to 2019. The CEE of Liaoning province declined the most over the study period, with the province that started out with the highest CEE becoming the lowest; Jilin province had a small but insignificant increase in CEE; and Heilongjiang province saw its CEE decline and then increase before finally becoming the most efficient province. All three fluctuated around 0.700 CEE between 2011 and 2016, with the CEE gap narrowing over the 13 years. Within the prefecture-level cities in 2019, Dalian, Harbin, Heihe, and Daqing had the highest CEE above 1, while Jilin, Baicheng, and Tieling had the smallest CEE at 0.379, 0.350, and 0.345, respectively. The top 10 CEE were recorded for large cities with large populations, except for Heihe, which had a CEE below 0.6. The reason for the high CEE in Heihe may have been the fact that it is a port city, generating more GDP with less CO_2_ emissions through import and export trade (Figure 4).

There was an increase in high-value PCR areas between 2006 and 2019, with PCR in 2019 showing a change from 0 tons to 33.74 million tons (Table A2). Jilin, Siping, Qiqihar, and Shenyang were the four prefecture-level cities with the largest PCR. Notably, Shenyang and Qiqihar, where heavy industry continues to predominate, consume more energy and produce more carbon emissions. Therefore, Qiqihar and Shenyang are key locations for reducing CO_2_ emissions, while other cities with a high PCR value are clustered around Shenyang and Changchun; therefore, more CO_2_-reduction efforts should be made in these regions. In the future, heavy industrial cities should promote the restructuring of their industries and technological innovation. At the same time, measures need to be taken to introduce advanced low-carbon technologies and strengthen inter-regional cooperation [58].

**Figure 5:**
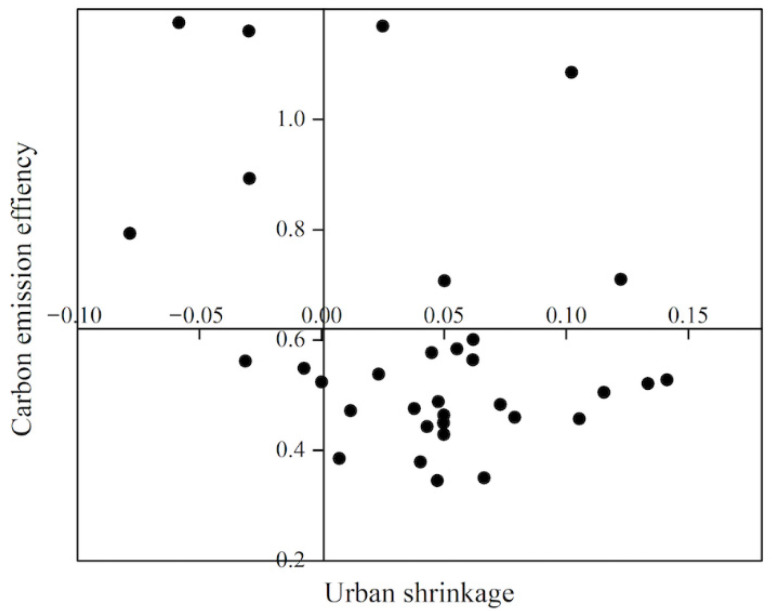
Analysis of urban shrinkage and carbon emission efficiency characteristics.

**Figure 6:**
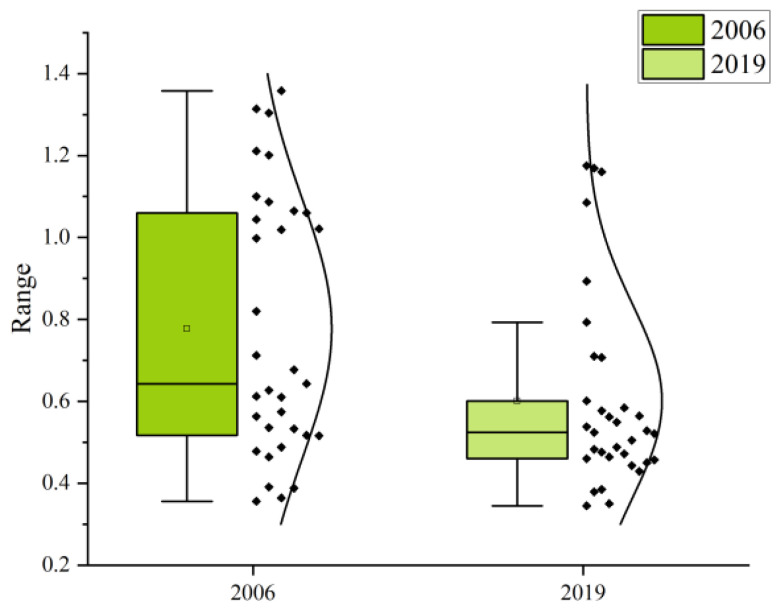
Box chart of carbon emission efficiency with scatter plot and distribution overlay.

We analyzed the relationship between urban shrinkage and carbon dioxide emission efficiency by using quadrant plots (Figure 5) and box plots (Figure 6). The intersection of the mean CEE (vertical axis) and urban shrinkage 0 (horizontal axis) of a sample of 33 cities in 2019 was taken as the coordinate origin to divide the four quadrants, the CEE and urban shrinkage of the study sample were compared and analyzed using scatter plots, and the spatial distribution of CEE was plotted to further analyze and summarize the spatial distribution characteristics of urban shrinkage and CEE.

In terms of contraction alone, population contraction is a serious problem in the northeast, with only six cities seeing their populations rise in the 2006–2019 period. The top five cities in terms of contraction were Yichun, Qitaihe, Jixi, Hegang, and Baishan, and the bottom five cities were Shenyang, Dalian, Panjin, Daqing, and Changchun.

Almost 70% of the cities are in the fourth quadrant, being contracting low-efficiency cities, and the vast majority of these are smaller than the medium-sized border cities. Only Harbin, Heihe, Jiamusi, and Jixi are contracting high-efficiency cities, but these four cities, with the exception of Harbin, continue to experience a steady decline in CEE. Dalian, Daqing, Changchun, and Shenyang are growth-oriented high-efficiency cities that, with the exception of Changchun, have also experienced a continuous decline in CEE. The box plot shows that the CEE data not only reflect an overall decrease, but also reveal that more than 75% of the data is concentrated in a much lower CEE range (0.4–0.6) compared with 2006 (0.5–1.1).

## 5. The Explanatory Factors of CO_2_ Emission Efficiency (CEE)

Table 4 reports the results of the baseline model regression for prefecture-level cities in northeast China. According to the Hausman test, the *p*-value is less than 0.1 and there is evidence to reject the original hypothesis and adopt estimation using a fixed effects panel model. In order to establish a reference point, we first present the basic OLS (Ordinary Least Squares) results in column 2. The OLS results indicate that urban shrinkage has a negative impact on CEE. The magnitude of this is −2.1927, which is significant at the 1% level of significance. However, this is a simple city-to-city comparison and further investigation incorporating city fixed effects is shown in column 3. The coefficient is still negative and significant at a 1% significance level. Also, the two-way fixed effects model (FE) as shown in column 4 also give the same estimation.

Model (c) in Table 4 is an individual and time double fixed effects model with a statistically significant negative coefficient for urban contraction at the 1% level after controlling for a range of other variables, which is consistent with the study’s findings [11]. For every 1% increase in urban shrinkage, CEE decreased by approximately 2.1458 percentage points, indicating that the dampening effect of urban shrinkage on economic output is greater than the saving effect of energy consumption, leading to an overall decrease in CEE.

In terms of the control variables, GDPP was positively correlated with CEE, consistent with the results of [32]. IS showed a negative coefficient of CEE, which was significant at the 5% level, mainly due to the weight of industry leading to more energy consumption, while most enterprises in the cities in the northeast could not effectively improve CEE due to the low level of development in energy saving and emission reduction technologies.

When it comes to the urban construction indictors, the regression coefficient for V was positive and passed the significance test at the 1% level of significance. This indicates that the economic benefits of urban sprawl in the northeast were greater than the increase in CO_2_ emissions. In contrast, the regression coefficient of EII was negative and passed the significance test at 1%. The absolute value of the regression coefficient of the V was much smaller than that of the EII, as most small and medium-sized cities are losing population, and an excessive increase in urban area will aggravate the unplanned layout problem of small towns, and greatly reduce the efficiency of land use for construction [59]. It will also greatly reduce the efficiency of land use for construction, resulting in a mismatch of resources, which will not be conducive to compact urban development [60]. The road area per capita (Road) have a positive coefficient, and it passes the significance test at the 1% level. This suggests that urban CEE in the northeast increases with increasing urban road area per capita. In line with the results of previous research [61], most small and medium-sized cities are in the early stages of urbanization, and an increase in Road would increase the operational efficiency of urban transport, and thus reduce carbon emissions. The correlation coefficient between Green and CEE is positive but not significant, suggesting that green space is not effective in increasing CEE, probably because people in the northeast still rely on motor vehicles to travel most of the time due to the long winters, and even if there are green space near residential areas, it still does not reduce motor vehicle use.

The results of models 1–3 in Table 5 show that the two variables, i.e., R&D and COMP, have a significant positive effect on CEE, both when analyzed separately and included in the analysis, indicating the robustness of this result. At this stage, cities in northeast China can still achieve CEE goals by exploiting the positive externalities of agglomeration economies. At the same time, the urban shrinkage coefficient is statistically significant at the 1% level, indicating that urban shrinkage has a strong negative effect on CEE. The absolute value of the urban shrinkage coefficient in Model (c) in Table 4 is larger than the estimated coefficients in models 1–3 in Table 5, which include the mediating variables and are consistent with the identification of mediating effects. In models (4) and (5), the urban shrinkage coefficients are significantly negative when R&D and COMP are considered as mediating variables. For a 1% increase in urban shrinkage, technological innovation capacity and urban compactness decrease by approximately 0.5344 and 1.2333 percentage points, respectively, suggesting that urban shrinkage significantly reduces the level of R&D and COMP. The above results are consistent with the criteria for mediating variables, and therefore it can be concluded that technological innovation capacity and urban compactness are the mediating variables of urban shrinkage affecting CEE.

## 6. Conclusions and Policy Implications

This study represents the first attempt to use gridded data (ODIAC) from northeast China to study the environmental impacts of prefecture-level municipalities. Unlike many national or provincial studies, this study used data from prefecture-level cities to provide more detailed information for policymakers. In addition, we investigated, for the first time, the impact of urban shrinkage, built-up area expansion, urban roads, and urban green space on CEE, and used a mediating effects model to identify the transmission mechanisms, which have often been overlooked in previous studies. The phenomenon of urban shrinkage has started to spread in some Chinese cities, arousing academic concerns. Exploring the energy and environmental changes caused by urban shrinkage and analyzing its pathways and mechanisms are important for the construction of ecological communities and the sustainable development of shrinking cities.

In this paper, the super-efficiency SBM model was used to analyze the CEE and PCR of northeast China for the 2006–2019 period. At the same time, the Tobit model and the mediating effects model were used to study the factors influencing CEE. The results show that: (1) the CEE of the cities in northeast China continues to be low, with an average CEE of 0.601 in 2019. Heilongjiang province recorded the best CEE of 0.724, followed by Jilin province (CEE of 0.688) and Liaoning province (CEE of 0.565), with the CEE of most contracting cities at relatively low levels; (2) with the development of new technologies, there is more scope for reducing carbon emissions in prefecture-level cities in the northeast. Qiqihar and Shenyang showed a larger PCR, so more responsibility for CO_2_ emission reduction should be allocated to these cities. The analysis of PCR is beneficial to the allocation of carbon emission reduction quotas; (3) urban shrinkage has a significant inhibitory effect on CEE, as CEE is mainly explained by the degree of urban shrinkage, population density, secondary industry share of GDP, road network density, and built-up area expansion index; and (4) urban shrinkage not only has a direct effect on CEE, but also has an impact on CEE through the pathways affecting R&D and urban compactness. Specifically, the current intensification of urban shrinkage in the northeast suppresses R&D inputs, which further undermines CEE as these two indicators decline.

CEE shows significant polarization in the cities in northeast China, most likely due to the different levels of urban shrinkage and economic development. Dalian, Harbin, Daqing, Shenyang, and Changchun have high CEEs, and as the most economically developed cities in the northeast, they also have the lowest urban shrinkage. In contrast, a large number of the cities shrinking the most had lower CEEs, and it is notable that the small and medium-sized cities in the Harbin–Changchun and Shenyang–Dalian agglomerations (e.g., Jilin, Siping, and Songyuan) did not benefit from the agglomeration effect, but were instead affected by the siphoning effect of the central cities, which increased their shrinkage. This shows that the spatial pattern of the division of labor and cooperation among the urban agglomerations in the northeast has not yet been formed, and the lack of coordination in planning the spatial layout of productivity has led to a conspicuous problem of duplication and an inefficient use of facilities.

At this stage, labor migration had a direct impact on local economic development, while stagnant economic development will further reduce the attractiveness of the cities, creating a vicious circle that is not conducive to a continuous improvement in CEE. The factors influencing CEE are also not limited to socio-economic factors such as GDPP and IS. There is also a noticeable inhibition of CEE also reflected V and expansion EII. A reasonable scale of urban shrinkage and planning is a compromise that reflects the virtuous battle between population growth, economic development, and carbon emission constraints. Therefore, to explore the objective facts of urban shrinkage, it is not only necessary to focus on the changes in their economic dimensions, but also to seek coping strategies from the perspective of predicting the overall construction scale and master planning for the future development of cities.

We propose the following recommendations for low-carbon urban development in northeast China. Firstly, our paper provides data on CEE and PCR in different cities. There are large spatial differences in CEE, and large cities should play the role of demonstration and radiation, and strengthen cooperation with neighboring cities. Meanwhile, the government should develop carbon emission reduction policies. According to the difference of PCR, different tasks of CO_2_ emission abatement should be assigned to cities. Second, despite the inclusion of urban development and construction factors, GDPP and IS still play an important role in influencing CEE. Therefore, when formulating policies, local governments should scientifically adjust the industrial structure of cities in order to ensure stable economic development, and reasonably forecast energy saving and carbon reduction targets in various sectors. Lastly, our results found that EII had marked effects on CEE; therefore, for cities that show a clear shrinkage trend, the expansion of urban built-up areas should be strictly controlled, making full use of existing construction space.

## 7. Limitations and Future Research Directions

This paper dissects the mechanisms by which urban shrinkage affects CEE. However, there are still some limitations which need to be addressed.

(1) The data selected for the super-efficient SBM model were chosen mainly based on their accessibility and convenience, and the precision of the study is still lacking; for example, the use of electricity consumption as a proxy for energy input does not take into account the energy consumption status of different cities; the use of the number of people employed ignores the impact of differences in the type and quality of labor in different industrial structures. Future research needs to be further refined in terms of data comprehensiveness and accuracy.

(2) Secondly, the annual data of this paper do not consider seasonal/monthly effects. As northeast China is a severely cold region, the traffic patterns are much different in the summer compared to the winter. A study of the different seasons or months is needed in further research.

(3) Finally, this paper did not consider the fact that a change in the head of state can serve as a control variable, since a new administration may have different attitudes toward energy efficiency, and thus, committing to public infrastructure projects that affect CEE compared to other states and within the same state before the administration is replaced.

## Figures and Tables

**Table 1:** Data characteristics of the input–output variables of northeast China in 2006–2019.

Variable	Unit	Minimum	Maximum	Mean	Standard Deviation	Observation
Capital	10^8^ dollars	21.822	5374.246	518.351	734.539	462
Labor	10^4^ persons	8.190	153.660	36.519	32.158	462
Electricity	10^6^ KW·h	147.010	92,077.740	7187.793	7967.104	462
GDP	10^8^ dollars	12.287	821.452	116.832	151.843	462
CO_2_	million tons	5.115	106.158	28.313	21.846	462

Note: Capital and GDP are converted into dollars ($) based on 2006 average exchange rate ($1 = 7.9735 Chinese yuan (CNY)).

**Table 2:** Descriptive statistics of inputs and outputs.

Variable	Unit	Minimum	Maximum	Mean	Standard Deviation	Observation
CEE		0.249	1.505	0.711	0.291	462
Shrink		−0.078	0.467	0.004	0.038	462
R&D	%	0.033	4.513	0.813	0.766	462
COMP	persons/km^2^	0.621	16.802	3.181	1.962	462
V	%	−28.260	20.340	1.952	3.94	462
EII	%	−0.159	0.210	0.02	0.039	462
Road	m^2^	3.240	71.660	10.29	5.323	462
Green	m^2^/person	1.97	264.100	43.73	32.88	462
GDPP	10^4^ dollars	0.078	2.107	0.405	0.271	462
IS	%	11.700	86.000	44.602	13.244	462

Note: R&D and GDPP are converted into US dollars ($) based on 2006 average exchange rate ($1 = 7.9735 Chinese yuan (CNY)).

**Table 3:** Correlation matrixes of inputs and outputs.

	Electricity	Labor	Capital	GDP	CO_2_
Electricity	1				
Labor	0.5943 ***	1			
Capital	0.6928 ***	0.7970 ***	1		
GDP	0.5850 ***	0.8443 ***	0.7727 ***	1	
CO_2_	0.5861 ***	0.8511 ***	0.7681 ***	0.7711 ***	1

Notes: *** denotes two-tailed significance at 1% level.

**Table 4:** The regression results.

Factors	(a) OLS	(b) FE	(c) FE
Shrink	−2.1927 ***	−2.0323 ***	−2.1458 ***
	(−6.43)	(−5.65)	(−5.87)
GDPP	0.0522 ***	0.0565 ***	0.0492 ***
	(8.78)	(8.68)	(6.22)
IS	−0.1113 **	−0.1164 **	−0.1178 ***
	(−2.08)	(−2.47)	(−2.59)
V	0.0053 **	0.0086	0.0089 *
	(2.06)	(2.21)	(2.13)
EII	−0.9562 *	−0.9442 *	−1.1074 **
	(−6.84)	(−3.63)	(−2.09)
Road	0.0962 **	0.0941 **	0.0851 **
	(4.10)	(2.51)	(2.30)
Green	−0.0133	−0.0167	−0.0181
	(−1.69)	(−1.96)	(−1.43)
Constant term	0.7187 ***	1.0043 ***	0.8644 ***
	(5.27)	(6.85)	(5.06)
City-fixed effects	No	Yes	Yes
Year-fixed effects	No	No	Yes
*p*-value of Hausman test		0.0000	0.0000
Number of observations	462	462	462
R-square	0.6334	0.4104	0.5836

***, **, and * represent significance levels of 1%, 5%, and 10%, respectively. The figures in brackets are probability values. The *t*-statistic is in brackets.

**Table 5:** Mediating effects test.

Factors	(1) CEE	(2) CEE	(3) CEE	(4) R&D	(5) COMP
Shrink	−1.9610 ***	−1.3076 ***	−1.2023 ***	−0.5344 ***	−1.2333 ***
	(−5.42)	(−3.70)	(−3.43)	(−2.66)	(−6.21)
R&D	0.0772 ***		0.0603 ***		
	(4.15)		(3.45)		
COMP		0.0617 ***	0.0588 ***		
		(8.58)	(8.22)		
GDPP	0.0458 ***	0.0655 ***	0.0621 ***	0.0097 **	−0.0239 ***
	(5.87)	(8.63)	(8.22)	(2.24)	(−5.55)
IS	−0.1226 ***	0.0007	−0.0086	0.0127	−0.1754 ***
	(−2.74)	(0.01)	(−0.20)	(0.51)	(−7.08)
V	0.0130 ***	0.0016	0.0052	−0.0119 ***	0.0107 ***
	(3.08)	(0.40)	(1.27)	(−5.19)	(4.69)
EII	−1.9397 ***	0.2693	−0.4459	2.4106 ***	−2.0379 ***
	(−3.48)	(0.52)	(−0.81)	(8.28)	(−7.06)
Road	0.0918 **	0.0192	0.0276	−0.0202	0.0968 ***
	(2.53)	(0.55)	(0.79)	(−0.99)	(4.82)
Green	−0.0148	0.0340	0.0306	0.0038	−0.0681 ***
	(−0.52)	(1.24)	(1.13)	(0.24)	(−4.34)
Constant term	0.8557 ***	0.1730	0.1987	0.0229	0.9538 ***
	(5.10)	(0.97)	(1.13)	(0.24)	(10.27)

*** and ** represent significance levels of 1% and 5%, respectively. The *t*-statistic is in brackets.

## Data Availability

Not applicable.

## Abbreviations

The following abbreviations are used in this manuscript:

CEE	CO_2_ emission efficiency
PCR	Potential CO_2_ emission reduction
R&D	Research and development expenditure
COMP	Urban population density
Road	Urban Road area per capita
Green	Green space area per capita
V	Average annual rate of expansion
EII	Expansion intensity index
GDPP	GDP per capita
IS	Industrial structure

## Appendix A

**Table A1:** CO_2_ emission efficiency (CEE) in northeast China during the 2006–2019 period.

Prefecture-Level City	2006	2007	2008	2009	2010	2011	2012	2013	2014	2015	2016	2017	2018	2019
Shenyang	0.998	1.001	1.098	1.084	0.937	0.953	0.937	0.927	0.997	0.896	0.857	0.708	0.751	0.793
Dalian	1.314	1.337	1.315	1.325	1.302	1.318	1.304	1.308	1.449	1.332	1.285	1.340	1.258	1.175
Anshan	1.211	1.179	1.195	1.167	1.154	1.137	1.100	1.070	1.039	0.678	0.693	0.416	0.477	0.538
Fushun	0.478	0.594	0.604	0.535	0.520	0.468	0.429	0.441	0.443	0.441	0.545	0.437	0.448	0.460
Benxi	0.563	0.613	0.649	0.697	1.012	0.716	0.822	1.042	0.762	1.014	1.052	0.463	0.473	0.483
Dandong	1.044	1.040	1.015	0.915	0.799	0.769	0.758	0.792	0.714	0.597	0.536	0.444	0.460	0.476
Jinzhou	1.087	1.098	1.106	1.117	1.102	0.803	0.744	0.756	0.782	0.775	1.014	0.739	0.602	0.464
Yingkou	0.536	0.606	0.614	0.639	0.566	0.554	0.462	0.493	0.488	0.521	0.554	0.445	0.484	0.524
Fuxin	0.356	0.337	0.344	0.317	0.368	0.375	0.349	0.370	0.377	0.392	0.460	0.395	0.442	0.488
Liaoyang	0.574	0.622	0.664	0.628	0.633	0.560	0.484	0.484	0.487	0.535	0.728	0.432	0.505	0.577
Panjing	1.019	1.009	0.733	0.695	0.489	0.637	0.600	0.667	0.654	0.605	0.552	0.445	0.504	0.562
Tieling	1.201	1.161	1.173	1.070	1.108	1.097	1.106	1.091	1.070	1.064	0.426	0.331	0.338	0.345
Chaoyang	0.712	0.796	0.799	0.698	0.658	0.665	0.554	0.632	0.636	0.696	0.479	0.407	0.440	0.472
Huludan	0.533	0.520	0.526	0.501	0.413	0.362	0.340	0.318	0.305	0.286	0.409	0.441	0.495	0.549
Changchun	0.820	0.668	0.681	0.648	0.565	0.711	0.565	0.781	0.758	0.857	0.744	0.688	0.791	0.893
Jilin	0.464	0.453	0.534	0.603	0.557	0.585	0.505	0.630	0.520	0.532	0.539	0.573	0.476	0.379
Siping	0.612	0.642	0.616	1.128	0.542	0.535	0.518	0.557	0.656	0.895	1.010	1.505	0.974	0.443
Liaoyuan	0.517	0.471	0.560	0.535	0.578	0.628	0.565	0.628	0.658	0.690	0.644	0.595	0.512	0.429
Tonghua	0.488	0.502	0.518	0.500	0.492	0.590	0.560	0.638	0.723	0.753	0.790	0.506	0.478	0.450
Baishan	0.516	0.500	0.446	0.428	0.465	0.435	0.417	0.507	0.516	0.516	0.538	0.538	0.497	0.457
Songyuan	1.100	1.038	1.058	1.111	1.065	1.143	1.131	1.061	1.018	0.943	1.034	0.901	0.643	0.385
Baicheng	0.627	0.782	0.780	0.804	1.073	1.079	1.044	1.064	1.059	0.783	0.524	0.423	0.386	0.350
Haerbin	1.065	0.775	0.794	0.725	0.816	1.003	0.767	0.743	0.972	1.033	1.164	1.169	1.169	1.169
Qiqihaer	0.610	0.589	0.609	0.568	0.546	0.620	0.621	0.520	0.526	0.565	0.780	0.894	0.739	0.584
Jixi	1.060	1.052	1.047	1.004	1.051	0.543	0.495	0.543	0.456	0.402	0.555	0.626	0.668	0.710
Hegang	0.391	0.366	0.391	0.384	0.385	0.365	0.341	0.388	0.306	0.252	0.403	0.403	0.454	0.505
Shaungyashan	0.364	0.328	0.333	0.336	0.364	0.561	0.542	0.571	0.499	0.413	0.421	0.467	0.515	0.564
Daqing	1.304	1.291	1.276	1.299	1.265	1.316	1.353	1.288	1.345	1.373	1.215	1.230	1.195	1.160
Yichun	0.677	0.451	0.424	0.401	0.383	0.362	0.291	0.309	0.329	0.322	0.419	0.445	0.487	0.528
Jiamusi	1.021	1.012	1.066	1.036	1.060	1.149	1.099	1.081	1.079	1.074	1.023	1.011	0.859	0.707
Qitaihe	0.388	0.374	0.391	0.414	0.458	0.462	0.478	0.339	0.267	0.249	0.379	0.431	0.476	0.521
Mudanjiang	0.643	0.650	0.667	0.616	0.709	0.751	0.814	1.040	1.146	1.086	1.037	1.020	0.810	0.601
Heihe	1.358	1.184	0.716	0.489	0.740	0.693	1.007	0.684	0.669	1.059	0.735	1.007	1.046	1.085

**Table A2:** Potential CO_2_ emission reduction (PCR) in northeast China during the 2006–2019 period (million tons).

Prefecture-Level City	2006	2007	2008	2009	2010	2011	2012	2013	2014	2015	2016	2017	2018	2019
Shenyang	12.85	16.52	11.87	10.09	17.25	19.62	20.96	22.59	22.95	23.21	22.29	30.00	22.07	22.58
Dalian	0.00	0.00	0.00	0.00	0.00	0.00	0.00	0.00	0.00	0.00	0.00	0.00	0.00	0.00
Anshan	0.00	0.00	0.00	0.00	0.00	0.00	0.00	0.00	0.00	1.68	2.19	9.37	12.88	7.70
Fushun	6.76	3.79	4.80	3.14	4.31	1.71	2.37	2.29	2.14	2.79	11.67	15.05	12.97	16.22
Benxi	1.27	1.17	0.96	0.43	0.00	1.10	0.00	0.00	0.00	0.00	0.00	4.79	5.43	5.11
Dandong	0.00	0.00	0.00	0.00	2.04	2.91	3.61	1.46	4.88	5.59	5.92	7.00	11.30	7.45
Jinzhou	0.00	0.00	0.00	0.00	0.00	0.94	0.00	0.00	3.39	3.70	0.00	6.92	12.41	9.73
Yingkou	8.13	6.89	6.55	7.23	13.69	14.54	4.52	4.12	3.71	2.81	14.99	16.70	18.26	16.33
Fuxin	5.99	7.30	8.20	8.75	6.18	5.34	5.62	5.24	5.03	4.98	10.24	10.85	12.63	10.80
Liaoyang	8.37	9.02	0.92	1.18	4.73	2.94	4.00	3.76	3.71	4.43	5.97	14.46	14.81	10.19
Panjing	0.00	0.00	0.21	0.00	1.27	5.20	5.80	6.77	7.08	8.41	8.83	9.77	7.98	8.08
Tieling	0.00	0.00	0.00	0.00	0.00	0.00	0.00	0.00	0.00	0.00	31.20	32.14	36.60	23.76
Chaoyang	6.01	2.91	4.71	7.53	9.35	8.79	9.07	10.08	6.65	6.54	18.04	18.54	23.14	18.56
Huludan	10.17	11.04	11.72	13.78	16.59	17.22	18.36	18.74	18.64	19.15	15.28	22.53	26.99	12.49
Changchun	13.22	20.24	24.13	18.75	34.72	35.53	37.65	22.80	31.97	28.56	37.64	52.19	52.66	15.69
Jilin	7.42	9.18	8.60	2.19	22.88	24.89	9.42	10.07	9.34	27.75	27.40	28.60	33.52	33.74
Siping	7.51	8.91	9.61	0.00	16.17	16.44	17.20	16.89	15.17	8.05	0.00	0.00	32.06	27.47
Liaoyuan	2.02	2.48	2.83	2.13	5.44	5.85	2.37	1.63	0.48	5.51	5.21	5.42	6.81	7.12
Tonghua	3.27	3.89	4.47	3.15	4.65	9.74	3.52	4.15	0.00	1.36	0.16	10.52	15.17	10.93
Baishan	6.44	7.36	7.46	7.58	6.68	6.37	6.94	6.20	5.91	5.74	10.64	10.97	12.64	11.65
Songyuan	0.00	0.00	0.00	0.00	0.00	0.00	0.00	0.00	0.00	0.74	0.00	4.68	19.57	17.17
Baicheng	5.50	1.21	1.32	0.62	0.00	0.00	0.00	0.00	0.00	2.47	10.62	11.13	14.39	11.98
Haerbin	1.07	12.86	13.15	10.88	18.16	11.02	4.08	7.72	7.69	3.99	0.00	0.00	0.00	0.00
Qiqihaer	11.34	13.51	13.97	16.39	18.52	17.28	19.15	19.11	19.00	17.59	10.54	1.25	34.46	30.37
Jixi	0.00	0.00	0.00	0.00	0.00	9.18	10.16	9.64	10.29	10.95	10.24	8.44	19.02	9.61
Hegang	8.44	9.20	9.20	10.35	12.77	13.37	14.05	13.92	14.85	15.70	13.67	12.91	16.22	11.23
Shaungyashan	12.81	13.48	14.08	15.06	18.61	18.79	19.54	19.67	19.51	21.99	18.88	23.97	29.27	16.90
Daqing	0.00	0.00	0.00	0.00	0.00	0.00	0.00	0.00	0.00	0.00	0.00	0.00	0.00	0.00
Yichun	4.85	7.35	7.72	8.40	7.31	7.75	8.65	8.57	8.30	8.62	10.75	9.79	11.72	7.66
Jiamusi	0.00	0.00	0.00	0.00	0.00	0.00	0.00	0.00	0.00	0.00	0.00	0.00	22.39	15.35
Qitaihe	8.16	8.61	9.20	9.35	9.12	8.83	10.85	11.68	12.92	13.20	11.69	14.44	11.66	10.96
Mudanjiang	3.11	2.83	3.07	3.76	9.93	8.93	8.81	0.00	0.00	0.00	0.00	0.00	25.84	19.28
Heihe	0.00	0.00	2.60	6.25	3.89	5.93	0.00	5.30	6.03	0.00	7.96	0.00	18.44	0.00
